# Supplementary material for: Direct observation of valley-polarized topological edge states in designer surface plasmon crystals
Source: Nat Commun. 2017 Nov 3;8:1304. doi: 10.1038/s41467-017-01515-2 (PMC5670222; doi:10.1038/s41467-017-01515-2)
Supplement: Supplementary file 1 — Supplementary Information [file 41467_2017_1515_MOESM1_ESM.pdf]

Supplementary Figures

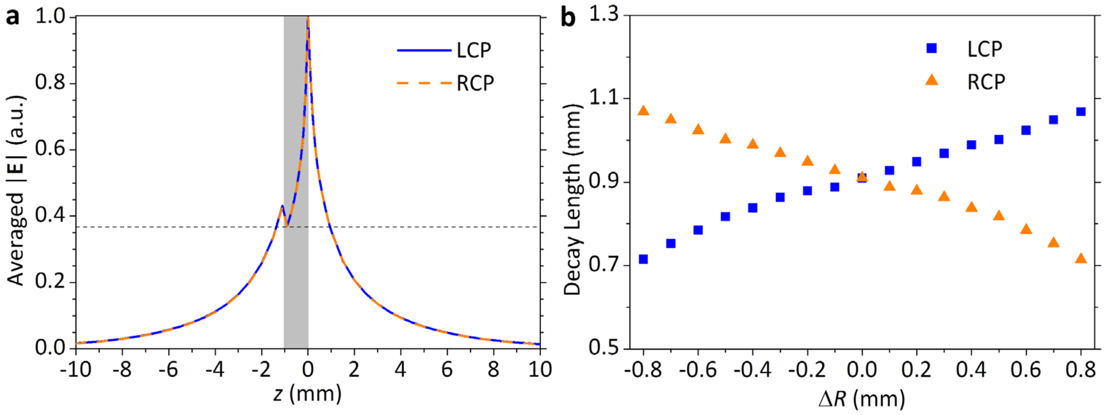

Supplementary Figure 1 | Confinement of the electric field near the surface at valley states. (a) Distribution of averaged amplitude of electric field |**E**| in the vertical (*z*) direction for LCP and RCP valley states when Δ*R* = 0. The maximum value is normalized to 1 and the dashed line indicates 1/*e*. (b) Decay length in the *z* direction for valley states of DSP crystals with different Δ*R*.

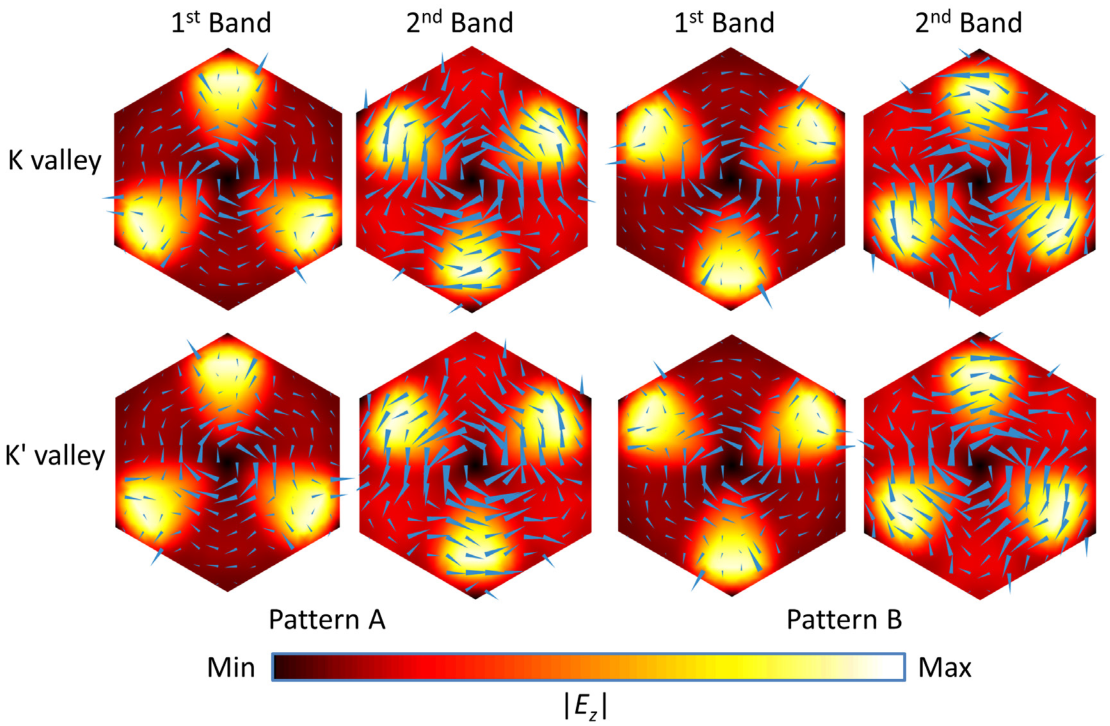

Supplementary Figure 2 | Eigenstates of the 1st and 2nd bands at K and K' valleys for Pattern A and Pattern B. The color represents amplitude of E_z component on *xy* plane 1 mm above the surface and the blue arrows represent the circulating energy flux. As can be observed, the energy flux at the K and K' valleys are exactly opposite for each band, respectively.

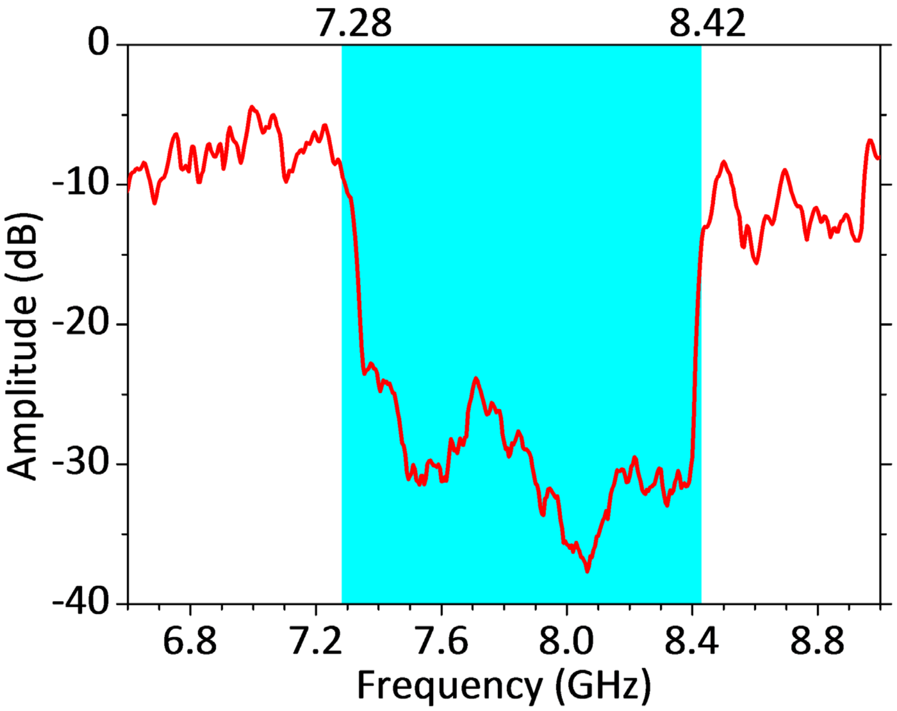

Supplementary Figure 3 | Experimental characterization of the bandgap. In experiments, a sample comprising only patterns A is used. The source is attached at the left side and the detector is placed at the right side of the sample. The measured frequency range of the bandgap is 7.28-8.42 GHz, denoted by the light blue shaded region.

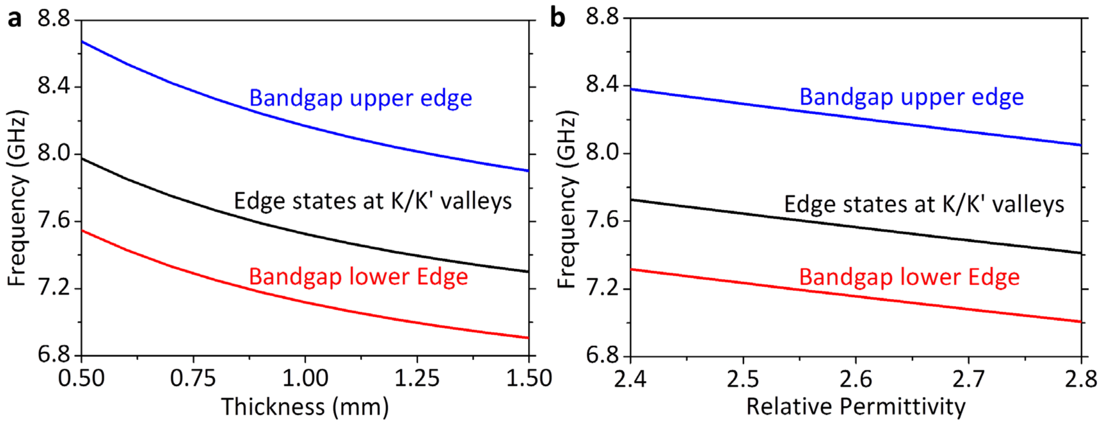

Supplementary Figure 4 | Bandgap of pattern A or pattern B with varying parameters. (a) The thickness is varied while the relative permittivity is kept constant at 2.65. (b) The relative permittivity is varied while the thickness is kept constant at 1 mm. It can be observed that the bandgap is only simply shifted for these possible errors in fabrications.

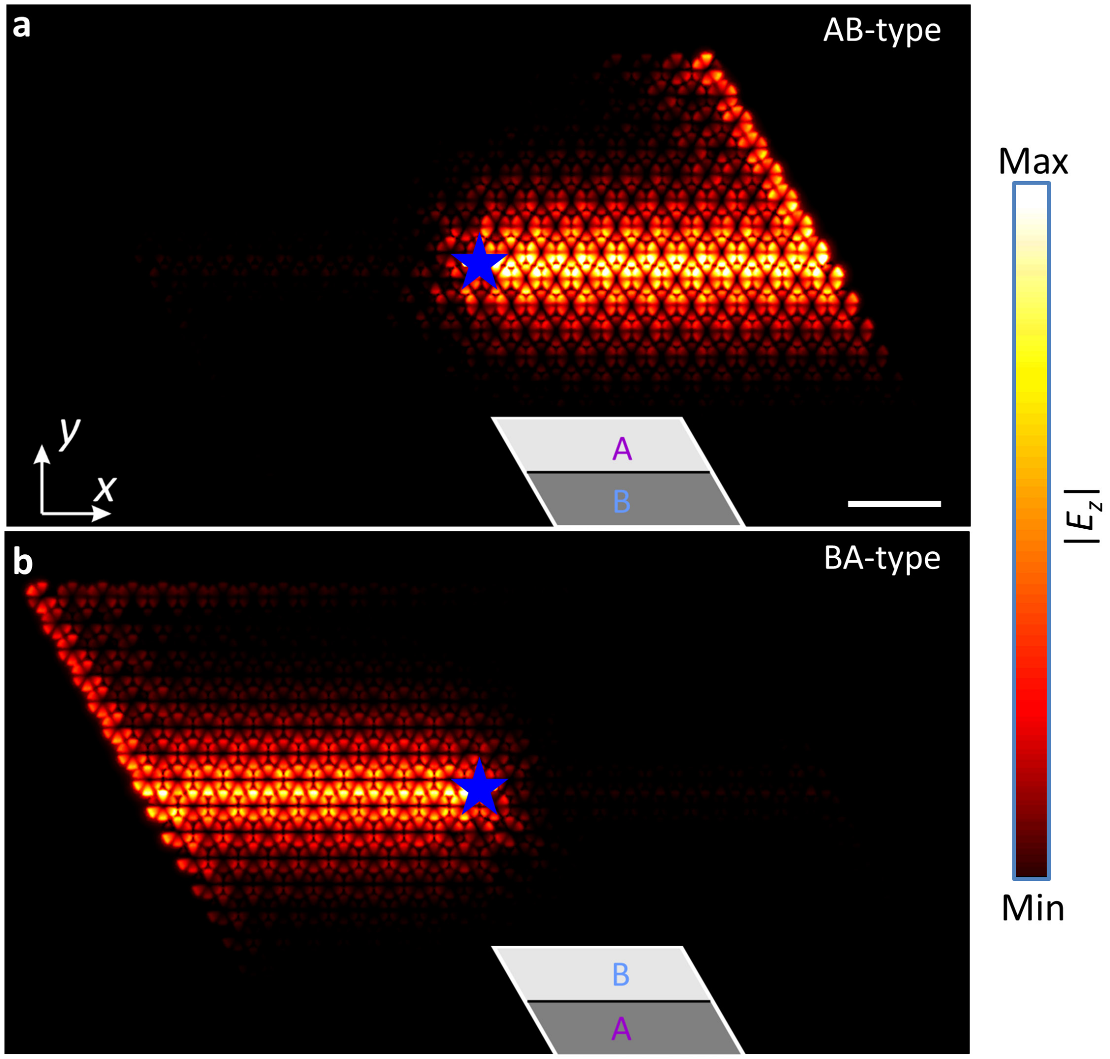

Supplementary Figure 5 | One-way propagation of K' valley-polarized edge states. The propagating direction of excited edge states on (a) AB-type and (b) BA-type domain walls agree with prediction from the band structure. The scale bar is 36 mm. The simulated frequency is 7.50 GHz. The color indicates the amplitude of E_z component. The blue star represents the source comprised of three phase-matched electrical dipoles whose phases are tuned such that they are matched with the phase of an edge state at the K' valley. The insets show the schematic diagrams of the domain walls.

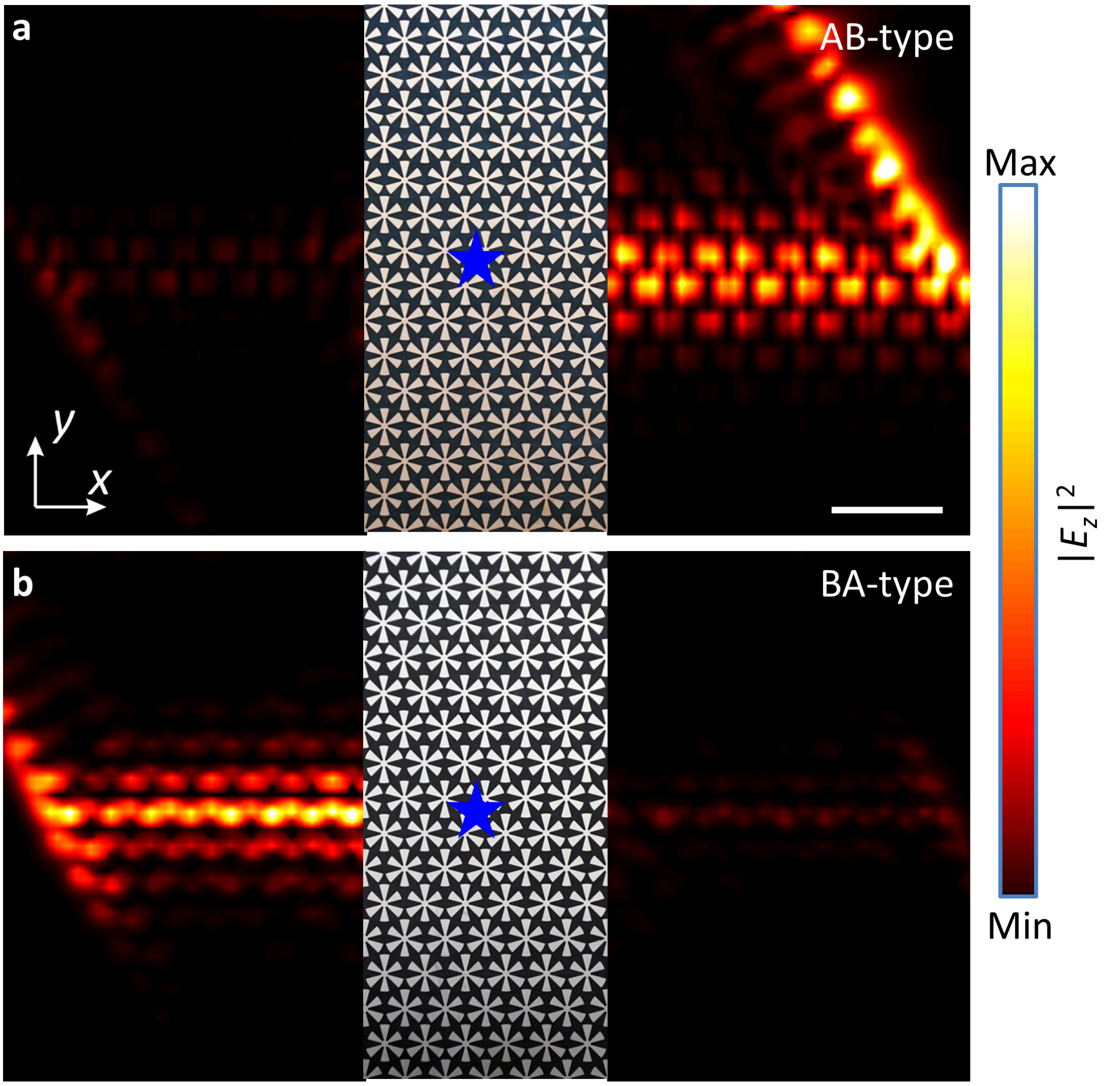

Supplementary Figure 6 | Experimental observations of one-way propagation of K' valley-polarized edge states. The area of scanned region at each side is 108×158 mm^2. The scale bar is 36 mm. The measured frequency is 7.50 GHz. The color indicates the squared amplitude of E_z component. The blue star represents the source comprised of three phase-matched monopole antennas whose phases are tuned using phase shifters such that they are matched with the phase of an edge state at the K' valley. The propagating direction of excited edge states on AB-type (a) and BA-type (b) domain walls agree with predictions from the band structure and simulations.

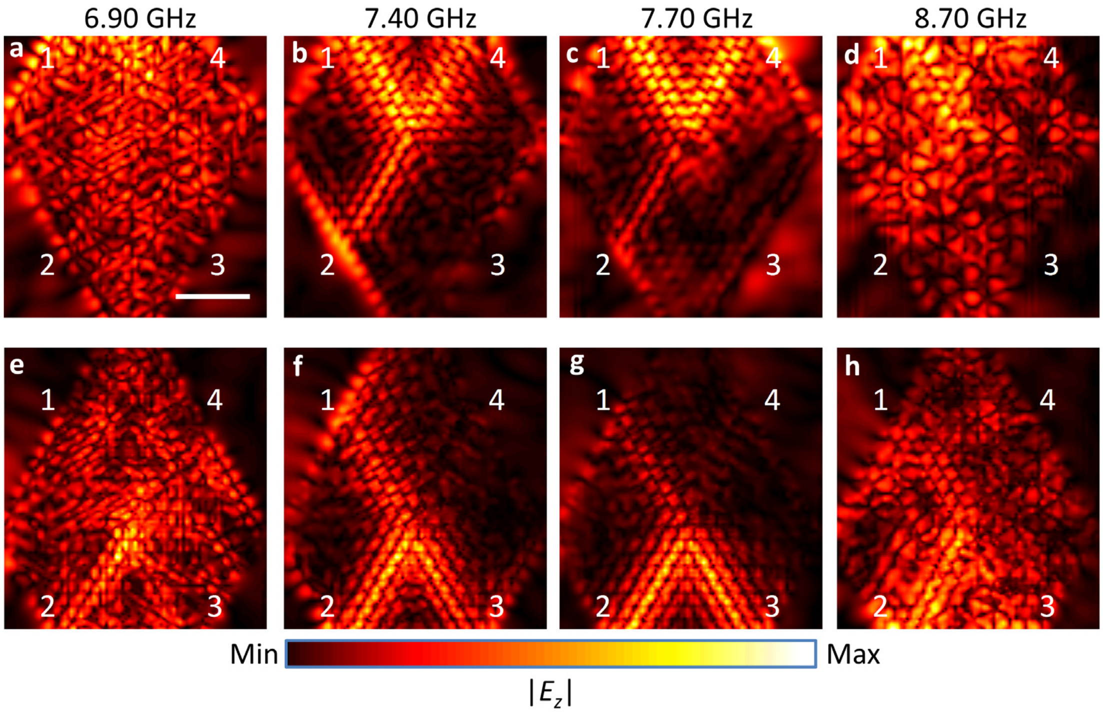

Supplementary Figure 7 | Measured near-field maps of E_z component above the beam splitter at different frequencies. The area of the scanned region is 205×220 mm^2. The scale bar is 60 mm. The source is attached at terminal 1 (a)-(d) or terminal 2 (e)-(h) in experiments. The measured frequencies are indicated in the figure. The color indicates the amplitude of E_z component.

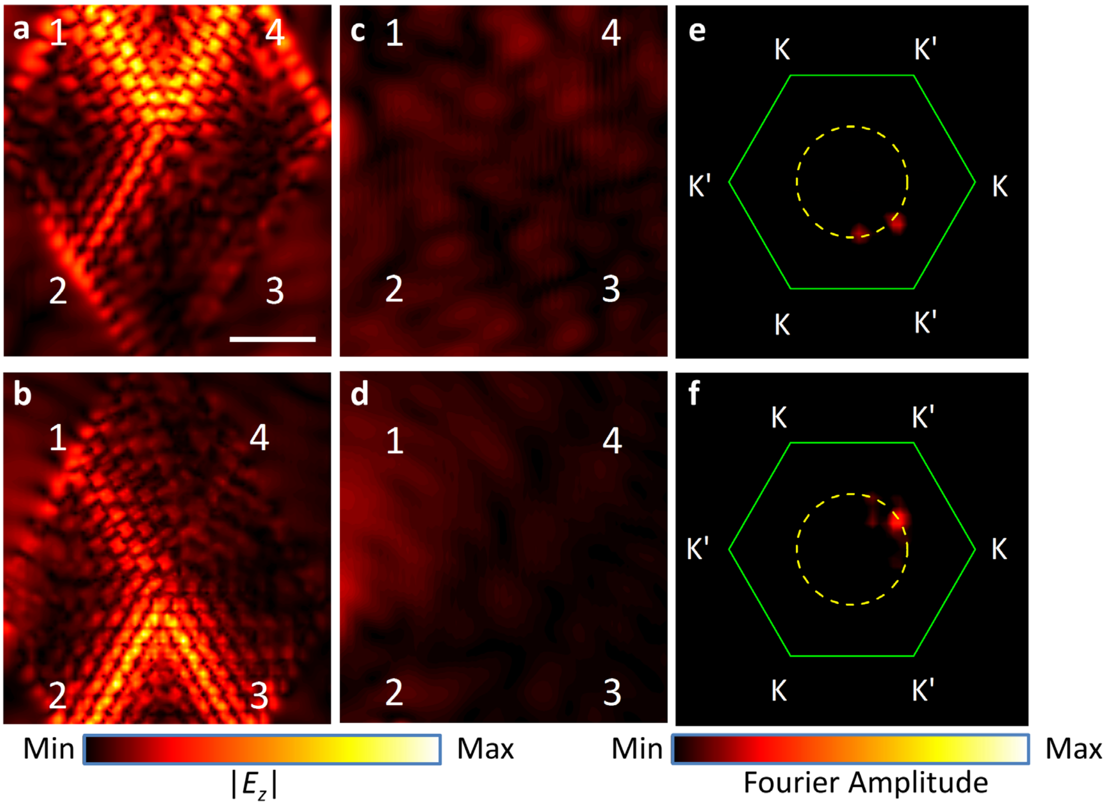

Supplementary Figure 8 | Comparison between measured fields when there are metallic patterns on the dielectric substrate or not. (a), (b) Measured field maps when there are metallic patterns on the substrate and the source is attached at terminal 1 (a) or terminal 2 (b), respectively. (c), (d) Measured field maps when there are no metallic patterns on the substrate and the source is also attached at terminal 1 (c) or terminal 2 (d), respectively. The area of the scanned region is 205×220 mm^2. The scale bar is 48 mm. The measured frequency is 7.50 GHz. The color indicates the amplitude of E_z component. (e), (f) Spatial Fourier transforms of the measured fields when there are no metallic patterns on the substrate. The green solid hexagons represent the FBZs of the DSP crystal and the yellow dashed circles represent the 7.50-GHz isofrequency contours of the light cone.

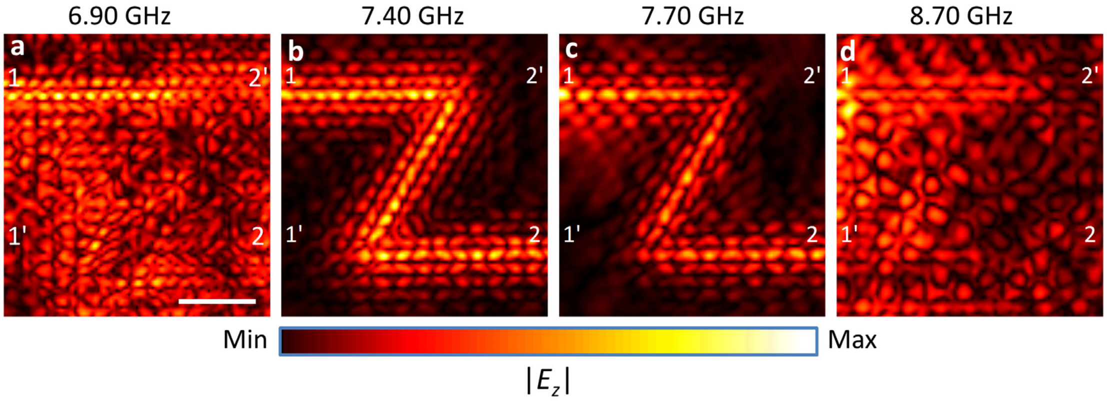

Supplementary Figure 9 | Measured near-field maps of E_z component above the Z-shaped waveguide at different frequencies. The area of the scanned region is 208×220 mm^2. The scale bar is 60 mm. The source is placed at terminal 1 in (a)-(d) and the measured frequencies are 6.90 GHz (a), 7.40 GHz (b), 7.70 GHz(c), and 8.70 GHz as indicated in the figure. The color indicates the amplitude of E_z component.

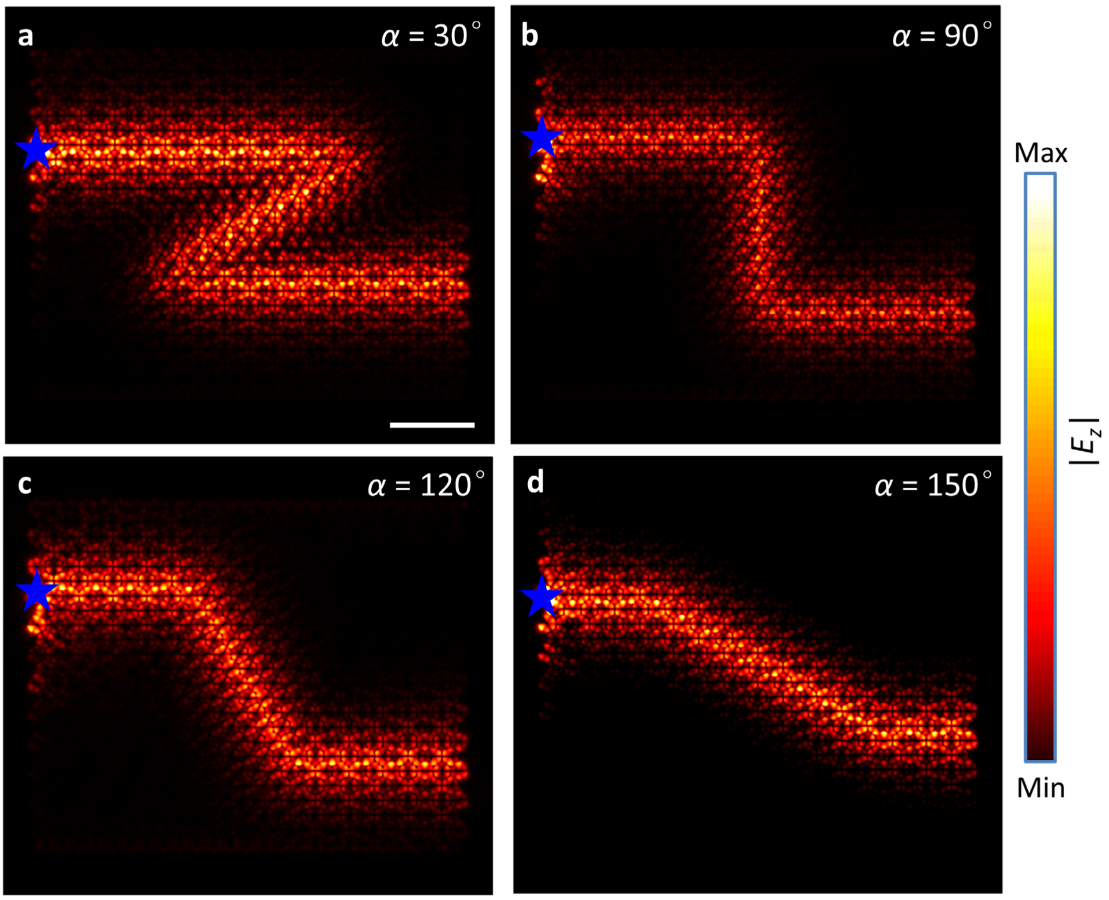

Supplementary Figure 10 | Edge state travels through Z-shaped waveguides with different corner angles *α*. (a) α = 30°, (b) α = 90°, (c) α = 120°, (d) α = 150°. When α = 30°, 90°, or 150°, the corner is incompatible with the triangular lattice. The scale bar is 60 mm. The color indicates the amplitude of E_z component. The blue star represents the source which is an electrical dipole. Seen from the field maps, there are no observable scattering losses at corners of the waveguides even when they are incompatible with the triangular lattice.

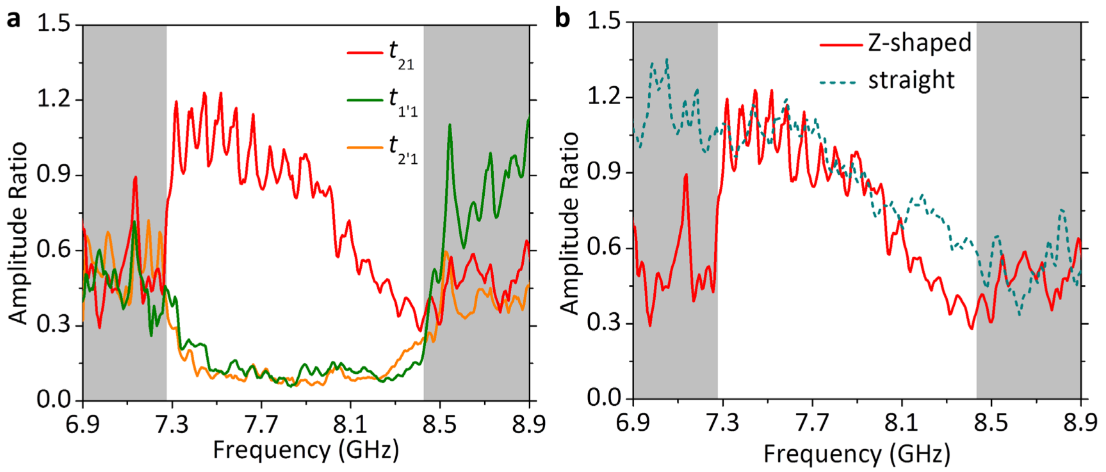

Supplementary Figure 11 | Measured transport of the edge state in topological waveguides. (a) Measured E_z amplitude ratio of the edge state in a Z-shaped waveguide at terminal 2 and two reference terminals 1' and 2' when the source is attached at terminal 1. The experimental bandgap is denoted by the unshaded region. The contrast in the bandgap suggests the edge state is well confined along the interface. (b) Comparison of the measured transport (E_z amplitude ratio) between the Z-shaped waveguide and a topological straight waveguide also comprised of the BA-type domain wall.

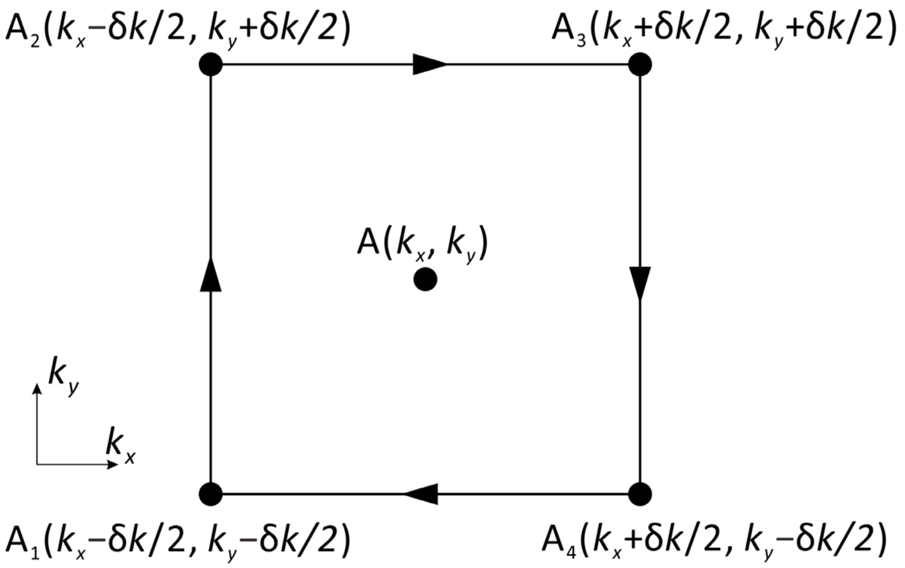

Supplementary Figure 12 | Schematic diagram of numerical calculations of Berry curvature. The arrows represent the direction (clockwise) of the path integral of the Berry connection around a certain point A in the reciprocal space.

Supplementary Notes

Supplementary Note 1

Construction of Effective Hamiltonian Model

Though there have been several effective Hamiltonians based on the **k·p** method for **H** field[1,2], in COMSOL Multiphysics **H** field is obtained by differentiating **E** field, and hence the quality of **H** field is much worse than **E** field. For benefit of numerical calculations, here we construct an effective Hamiltonian for **E** field to model the topological phase transition based on the **k·p** method.

We first define an inner product between **E** fields of two eigenstates $\mathbf{E}_1(\mathbf{r})$ and $\mathbf{E}_2(\mathbf{r})$ in the designer surface plasmon (DSP) crystal

$$\left(\mathbf{E}_{1,}\mathbf{E}_2\right) = \int_V \varepsilon(\mathbf{r})\mathbf{E}_1^*(\mathbf{r}) \cdot \mathbf{E}_2(\mathbf{r})\mathrm{d}^3\mathbf{r}\,, \tag{1}$$

in which V is the volume of a unit cell of the DSP crystal, $\varepsilon(\mathbf{r})$ representing the distribution of relative permittivity in V, and * denoting complex conjugate. It is easy to verify the definition satisfies the three requirements of an inner product, that is, $(\mathbf{E}_1, \mathbf{E}_1) \geq 0$, $(\mathbf{E}_1, \mathbf{E}_2) = (\mathbf{E}_2, \mathbf{E}_1)^*$, $(\mathbf{E}_1, \mathbf{E}_2 + \mathbf{E}_3) = (\mathbf{E}_1, \mathbf{E}_2) + (\mathbf{E}_1, \mathbf{E}_3)$.

The governing wave equation in the frequency domain for the electric field $\mathbf{E}(\mathbf{r})$ is

$$\nabla \times \nabla \times \mathbf{E} = \frac{\omega^2}{\mathrm{c}^2}\varepsilon(\mathbf{r})\mathbf{E}\,, \tag{2}$$

in which ω is the angular frequency, c the speed of light in vacuum, $\varepsilon(\mathbf{r})$ is the relative permittivity. After all the eigenstates at a point $\mathbf{k} = \mathbf{k}_0$ in the reciprocal space have been known and suppose one of them with angular frequency $\omega_{j,\mathbf{k}_0}$ could be represented as $\mathbf{E}_{n,\mathbf{k}0}(\mathbf{r}) = \mathbf{u}_{n,\mathbf{k}_0}(\mathbf{r})e^{i\mathbf{k}_0\cdot\mathbf{r}}$, in which n denotes the band index and

$\mathbf{u}_{n,\mathbf{k}_0}(\mathbf{r})$ is a periodic function, the nth eigenstate near $\mathbf{k}_0$ then could be expanded as[3]

$$\mathbf{E}_{n,\mathbf{k}}(\mathbf{r}) = \mathbf{u}_{n,\mathbf{k}}(\mathbf{r})e^{i\mathbf{k}\cdot\mathbf{r}} = \sum_j A_{n,j}(\mathbf{k})e^{i\delta\mathbf{k}\cdot\mathbf{r}}\mathbf{E}_{j,\mathbf{k}_0}(\mathbf{r}), \tag{3}$$

in which the unknown periodic function $\mathbf{u}_{n,\mathbf{k}}(\mathbf{r})$ is expanded as a linear combination of $\mathbf{u}_{j,\mathbf{k}0}(\mathbf{r})$ and $\delta\mathbf{k} = \mathbf{k}-\mathbf{k}_0$, and $A_{n,j}(\mathbf{k})$ are the expansion coefficients. For convenience, we could assume that all the eigenstates have been normalized such that[4]

$$(\mathbf{E}_{l,\mathbf{k}_0}, \mathbf{E}_{j,\mathbf{k}_0}) = \delta_{lj}, \tag{4}$$

in which δ_{lj} is the Kronecker delta function. Substituting Supplementary Eq. (3) into Supplementary Eq. (2), we have

$$\begin{aligned}&\sum_j A_{n,j}\{-\delta\mathbf{k}\times(\delta\mathbf{k}\times\mathbf{E}_{j,\mathbf{k}_0}) + i[\nabla\times(\delta\mathbf{k}\times\mathbf{E}_{j,\mathbf{k}_0}) + \delta\mathbf{k}\times(\nabla\times\mathbf{E}_{j,\mathbf{k}_0})]\} \\ &= \sum_j \frac{\omega_{j,\mathbf{k}_0}^2 - \omega_{n,\mathbf{k}}^2}{\mathrm{c}^2}\varepsilon\mathbf{E}_{j,\mathbf{k}_0}\end{aligned}. \tag{5}$$

Utilizing the orthonormal property of the eigenstates stated in Supplementary Eq. (4) and noticing that the fields exponentially decay in z direction, Supplementary Eq. (5) could be simplified to a number of algebra equations

$$\sum_j [\delta\mathbf{k}\cdot\mathbf{p}_{lj} + \delta\mathbf{k}^{\mathrm{T}}q_{lj}\delta\mathbf{k}]A_{n,j} = \frac{\omega_{n,\mathbf{k}}^2 - \omega_{l,\mathbf{k}_0}^2}{\mathrm{c}^2}A_{n,l}, \tag{6}$$

in which the vectors p_{lj} and the tensors q_{lj} are defined as[1]

$$\mathbf{p}_{lj} = -i\int_{\mathrm{V}}[(\nabla\times\mathbf{E}_{l,\mathbf{k}_0}^*)\times\mathbf{E}_{j,\mathbf{k}_0} + \mathbf{E}_{l,\mathbf{k}_0}^*\times(\nabla\times\mathbf{E}_{j,\mathbf{k}_0})]\mathrm{d}V, \tag{7}$$

$$q_{lj} = \int_{\mathrm{V}}(\mathbf{E}_{l,\mathbf{k}_0}^*\cdot\mathbf{E}_{j,\mathbf{k}_0} - \mathbf{E}_{l,\mathbf{k}_0}^*\otimes\mathbf{E}_{j,\mathbf{k}_0})\mathrm{d}V, \tag{8}$$

and it could be verified that $\mathbf{p}_{lj}$ and q_{lj} are Hermitian, that is, $\mathbf{p}_{lj}^* = \mathbf{p}_{jl}$, $q_{lj}^\dagger = q_{jl}$. Therefore, we could define a Hermitian matrix L

$$L_{lj} = \mathrm{c}^2\delta\mathbf{k}\cdot\mathbf{p}_{lj} + \mathrm{c}^2\delta\mathbf{k}^{\mathrm{T}}q_{lj}\delta\mathbf{k}, \tag{9}$$

and Supplementary Eq. (6) then could be reorganized as

$$\sum_j L_{lj} A_{n,j} = (\omega_{n,\mathbf{k}}^2 - \omega_{l,\mathbf{k}_0}^2) A_{n,l} . \tag{10}$$

Then we construct a lowest-order effective Hamiltonian at the K valley by only including the first two bands. With this approximation, Supplementary Eq. (10) is reduced to

$$L_{11} A_1 + L_{12} A_2 = (\omega_{\mathbf{k}}^2 - \omega_{1,\mathbf{k}_0}^2) A_1 , \tag{11}$$

$$L_{12}^* A_1 + L_{22} A_2 = (\omega_{\mathbf{k}}^2 - \omega_{2,\mathbf{k}_0}^2) A_2 , \tag{12}$$

in which we have omitted the index n (= 1, 2) of $A_{n,j}$ and $\omega_{n,\mathbf{k}}$, and the angular frequencies could be parameterized to

$$\omega_{1,\mathbf{k}_0} = \omega_{\mathrm{V}} + \Xi , \tag{13}$$

$$\omega_{2,\mathbf{k}_0} = \omega_{\mathrm{V}} - \Xi , \tag{14}$$

$$\omega_{\mathbf{k}} = \omega_{\mathrm{V}} + \delta\omega , \tag{15}$$

in which 1 and 2 are LCP and RCP states at the K valley, respectively, and this representation is swapped at the K' valley since the energy flux is opposite for the same band as shown in Supplementary Fig. 2. In Supplementary Eqs. (13)-(15), ω_{V} is the mean angular frequency of the 1st and 2nd bands at valleys, and is slightly different from the Dirac frequency ω_{D} when ΔR is nonzero. Inserting Supplementary Eqs. (13)-(15) and Supplementary Eq. (9) into Supplementary Eqs. (11) and (12), and neglecting higher-order terms, we arrive at

$$\delta\mathbf{k} \cdot \mathbf{p}_{11} A_1 + \delta\mathbf{k} \cdot \mathbf{p}_{12} A_2 = \frac{2\omega_{\mathrm{V}}}{\mathrm{c}^2} (\delta\omega - \Xi) A_1 , \tag{16}$$

$$\delta\mathbf{k} \cdot \mathbf{p}_{12}^* A_1 + \delta\mathbf{k} \cdot \mathbf{p}_{22} A_2 = \frac{2\omega_{\mathrm{V}}}{\mathrm{c}^2} (\delta\omega + \Xi) A_2 , \tag{17}$$

which could be rearranged as

$$(\frac{c^2}{2\omega_V}\delta\mathbf{k}\cdot\mathbf{p}_{11}+\Xi)A_1+\frac{c^2}{2\omega_V}\delta\mathbf{k}\cdot\mathbf{p}_{12}A_2=\delta\omega A_1, \quad (18)$$

$$\frac{c^2}{2\omega_V}\delta\mathbf{k}\cdot\mathbf{p}_{12}^*A_1+(\frac{c^2}{2\omega_V}\delta\mathbf{k}\cdot\mathbf{p}_{22}-\Xi)A_2=\delta\omega A_2. \quad (19)$$

Therefore, we could define an effective Hamiltonian near K valley

$$H_K=\begin{bmatrix}\frac{c^2}{2\omega_V}\delta\mathbf{k}\cdot\mathbf{p}_{11}+\Xi & \frac{c^2}{2\omega_V}\delta\mathbf{k}\cdot\mathbf{p}_{12}\\ \frac{c^2}{2\omega_V}\delta\mathbf{k}\cdot\mathbf{p}_{12}^* & \frac{c^2}{2\omega_V}\delta\mathbf{k}\cdot\mathbf{p}_{22}-\Xi\end{bmatrix}, \quad (20)$$

and Supplementary Eq. (18) then could be written as

$$H_K A=\delta\omega A, \quad (21)$$

in which the vector A = [A_1; A_2]. In order to reveal the relations between **p** parameters, we numerically calculate them at the K valley for ΔR = 0.5 mm and the result is (in unit of m^{-1})

$$\mathbf{p}_{11}=\mathbf{p}_{22}=(0,0), \quad (22)$$

$$\mathbf{p}_{12}=(21.350-53.390i,-53.381-21.345i), \quad (23)$$

with a tolerance of 10^{-3}, which suggests the following relations

$$(\mathrm{Re}\,\mathbf{p}_{12})_x(\mathrm{Re}\,\mathbf{p}_{12})_y+(\mathrm{Im}\,\mathbf{p}_{12})_x(\mathrm{Im}\,\mathbf{p}_{12})_y=0, \quad (24)$$

$$(\mathrm{Re}\,\mathbf{p}_{12})_x^2+(\mathrm{Im}\,\mathbf{p}_{12})_x^2=(\mathrm{Re}\,\mathbf{p}_{12})_y^2+(\mathrm{Im}\,\mathbf{p}_{12})_y^2. \quad (25)$$

Therefore, we could introduce two real orthogonal vectors with the same norm

$$\boldsymbol{\alpha}_x=((\mathrm{Re}\,\mathbf{p}_{12})_x,-(\mathrm{Im}\,\mathbf{p}_{12})_x,0), \quad (26)$$

$$\boldsymbol{\alpha}_y=((\mathrm{Re}\,\mathbf{p}_{12})_y,-(\mathrm{Im}\,\mathbf{p}_{12})_y,0), \quad (27)$$

$$|\boldsymbol{\alpha}_1|=|\boldsymbol{\alpha}_2|=\alpha, \quad (28)$$

and the effective Hamiltonian then could be expressed as

$$H_{\mathrm{K}}=\begin{bmatrix}\Xi & \frac{c^2}{2\omega_{\mathrm{V}}}\delta\mathbf{k}\cdot(\mathrm{Re}\,\mathbf{p}_{12}+i\,\mathrm{Im}\,\mathbf{p}_{12})\\ \frac{c^2}{2\omega_{\mathrm{V}}}\delta\mathbf{k}\cdot(\mathrm{Re}\,\mathbf{p}_{12}-i\,\mathrm{Im}\,\mathbf{p}_{12}) & -\Xi\end{bmatrix}$$
$$=\frac{c^2}{2\omega_{\mathrm{V}}}\delta k_x[(\mathrm{Re}\,\mathbf{p}_{12})_x\sigma_x-(\mathrm{Im}\,\mathbf{p}_{12})_x\sigma_y]+ \quad . \tag{29}$$
$$\frac{c^2}{2\omega_{\mathrm{V}}}\delta k_y[(\mathrm{Re}\,\mathbf{p}_{12})_y\sigma_x-(\mathrm{Im}\,\mathbf{p}_{12})_y\sigma_y]+\Xi\sigma_z$$
$$=\frac{c^2}{2\omega_{\mathrm{V}}}\delta k_x\boldsymbol{\alpha}_x\cdot\boldsymbol{\sigma}+\frac{c^2}{2\omega_{\mathrm{V}}}\delta k_y\boldsymbol{\alpha}_y\cdot\boldsymbol{\sigma}+\Xi\sigma_z$$

This form of the effective Hamiltonian suggests that if we rotate the axis in the pseudospin space spanned by the orbital freedom such that x and y axes parallel to $\boldsymbol{\alpha}_x$ and $\boldsymbol{\alpha}_y$, the Hamiltonian will be casted into

$$H_{\mathrm{K}}=\frac{c^2}{2\omega_{\mathrm{V}}}\alpha\delta k_x\sigma_x+\frac{c^2}{2\omega_{\mathrm{V}}}\alpha\delta k_y\sigma_y+\Xi\sigma_z\,. \tag{30}$$

After a simple parameterization

$$\frac{c^2}{2\omega_{\mathrm{V}}}\alpha=v_{\mathrm{D}}\,, \tag{31}$$

$$\Xi=v_{\mathrm{D}}\Delta_{\mathrm{p}}\,. \tag{32}$$

Supplementary Eq. (30) becomes

$$H_{\mathrm{K}}=v_{\mathrm{D}}\delta k_x\sigma_x+v_{\mathrm{D}}\delta k_y\sigma_y+v_{\mathrm{D}}\Delta_{\mathrm{p}}\sigma_z\ , \tag{33}$$

which is the desired effective Hamiltonian near K valley. It is easy to verify that the value of v_{D} calculated from Eq. (31) is $5.45\times10^7\ \mathrm{m\cdot s^{-1}}$, which quantitatively agree with the value $5.42\times10^7\ \mathrm{m\cdot s^{-1}}$ obtained by fitting the numerical band structure and the deviation is smaller than 1%.

Then we could derive the effective Hamiltonian near K' valley using time-reversal operation. The time-reversal operator could be generally expressed as T

= UC, in which U is a unitary operator and C is the complex conjugation operator[5]. Because we choose the representation A_{R} = [1; 0], A_{L} = [0; 1] at the K valley and A_{L} = [1; 0], A_{R} = [0; 1] at the K' valley, U should be an identity matrix and hence $T = C$. Therefore, the effective Hamiltonian near K' valley is then[1,2]

$$\begin{aligned} H_{\mathrm{K'}}(\delta\mathbf{k}) &= TH_{\mathrm{K}}(-\delta\mathbf{k})T^{-1} \\ &= -v_{\mathrm{D}}\delta k_x\sigma_x + v_{\mathrm{D}}\delta k_y\sigma_y + v_{\mathrm{D}}\Delta_{\mathrm{p}}\sigma_z \end{aligned} . \tag{34}$$

Supplementary Note 2

Analytic Calculation of Berry Curvature

From the effective Hamiltonian near K valley as shown in Supplementary Eq. (33), the normalized eigenvector of the 1st band is

$$A_1 = \frac{1}{\sqrt{1+\dfrac{(\Delta_{\mathrm{p}}-\sqrt{\Delta_{\mathrm{p}}^2+\delta k_x^{\,2}+\delta k_y^{\,2}})^2}{\delta k_x^{\,2}+\delta k_y^{\,2}}}}\begin{bmatrix} \dfrac{\Delta_{\mathrm{p}}-\sqrt{\Delta_{\mathrm{p}}^2+\delta k_x^{\,2}+\delta k_y^{\,2}}}{\delta k_x + i\delta k_y} \\ 1 \end{bmatrix}, \tag{35}$$

with eigenfrequency

$$\delta\omega = -\sqrt{\Delta_{\mathrm{p}}^2+\delta k_x^{\,2}+\delta k_y^{\,2}} . \tag{36}$$

Therefore, the Berry connection could be calculated according to the conventional definition

$$\mathbf{B} = i\left(A_1, \nabla_{\mathbf{k}} A_1\right) = \frac{1+\dfrac{\Delta_{\mathrm{p}}}{\sqrt{\Delta_{\mathrm{p}}^2+\delta k^2}}}{2\delta k^2}(-\delta k_y\mathbf{e}_x + \delta k_x\mathbf{e}_y), \tag{37}$$

and then the Berry curvature (z component) is

$$\Omega_{\mathrm{K}} = \frac{\partial B_y}{\partial k_x} - \frac{\partial B_x}{\partial k_y} = \frac{\Delta_{\mathrm{p}}}{2(\delta k^2+\Delta_{\mathrm{p}}^{\,2})^{3/2}} . \tag{38}$$

The Berry curvature near K’ valley could be similarly calculated and the result is

$$\Omega_{\mathrm{K}'} = -\frac{\varDelta_{\mathrm{p}}}{2(\delta k^2 + \varDelta_{\mathrm{p}}^{\ 2})^{3/2}} \quad . \tag{39}$$

Supplementary Note 3

Derivation of Edge States from Effective Hamiltonian Model

In order to analytically derive the edge states, we consider a continuum model[6]. For simplicity but without loss of generality, we suppose that a homogenous domain wall is oriented along the x axis and formed by two DSP crystals with the parameter $\varDelta_{\mathrm{p}}$ ($\varDelta_{\mathrm{p}} > 0$) for $y > 0$ and $-\varDelta_{\mathrm{p}}$ for $y < 0$. As a result, the Valley Chern number at the K valley is +1/2 for $y > 0$ and −1/2 for $y < 0$, corresponding to a BA-type domain wall. The effective Hamiltonian around K valley is

$$H_{\mathrm{K}} = \begin{cases} v_{\mathrm{D}}\delta k_x\sigma_x + v_{\mathrm{D}}\delta k_y\sigma_y - \varDelta_{\mathrm{p}}\sigma_z & (y > 0) \\ v_{\mathrm{D}}\delta k_x\sigma_x + v_{\mathrm{D}}\delta k_y\sigma_y + \varDelta_{\mathrm{p}}\sigma_z & (y < 0) \end{cases} \quad . \tag{40}$$

Then we consider an eigenstate of the Hamiltonian represented by a vector A with eigenfrequency $\delta\omega$. Substituting the variable δk_y with the corresponding operator $-i\partial_y$, the governing equation in $y < 0$ is then

$$(v_{\mathrm{D}}\delta k_x\sigma_x - v_{\mathrm{D}}i\partial_y\sigma_y + v_{\mathrm{D}}\varDelta_{\mathrm{p}}\sigma_z)A = \delta\omega A \tag{41}$$

Then we suppose that A is an edge state which has the form in $y < 0$

$$A = \Phi e^{i\delta k_x x + \kappa y} \quad , \tag{42}$$

in which Φ is a spinor independent of coordinates. Substituting Supplementary Eq. (42) back to Supplementary Eq. (41) leads to

$$(v_{\mathrm{D}}\delta k_x\sigma_x - v_{\mathrm{D}}i\kappa\sigma_y + v_{\mathrm{D}}\varDelta_{\mathrm{p}}\sigma_z)\Phi = \delta\omega\Phi \quad . \tag{43}$$

For an edge state travelling along the domain wall, the band inversion between the two domains imposes a further requirement[7]

$$\Phi = \sigma_x \Phi . \tag{44}$$

Inserting Supplementary Eq. (43) into Supplementary Eq. (44), we have

$$v_{\mathrm{D}} \delta k_x \Phi + v_{\mathrm{D}} (\Delta_{\mathrm{p}} - \kappa) \sigma_z \Phi = \delta\omega \Phi , \tag{45}$$

which is identical and hence the expression in the round brackets should be zero. We then arrive at desired relations in $y < 0$

$$\kappa = \Delta_{\mathrm{p}} , \tag{46}$$

$$\delta\omega = v_{\mathrm{D}} \delta k_x . \tag{47}$$

Similar arguments will give the relations in $y > 0$

$$\kappa = -\Delta_{\mathrm{p}} , \tag{48}$$

$$\delta\omega = v_{\mathrm{D}} \delta k_x . \tag{49}$$

Therefore, the edge state has the form

$$A = \Phi e^{i\delta k_x x - \Delta_{\mathrm{p}} |y|} , \tag{50}$$

which travels along $+x$ direction consistent with the numerical band structure (BA-type domain wall) and the decay character in the bulk is then $|E_z| \propto e^{-\Delta_{\mathrm{p}}|y|}$. It should be noted that when $\delta k_x = 0$, we have $\delta\omega = 0$ according to Supplementary Eq. (47) or Supplementary Eq. (49), hence the angular frequency of the edge states is ω_{V} at valleys, in the middle of the 1st and 2nd bands. Similarly, the edge states around the other valley or of the other domain wall could be derived, and the results all agree with the bulk-boundary correspondence and numerical band structures.

Supplementary Note 4

Numerical Calculation of Berry Curvature

The Berry potential of a state at the ith band is defined as[8]

$$\mathbf{B}(\mathbf{k}) = i(\mathbf{u}_{i,\mathbf{k}}, \nabla_{\mathbf{k}}\mathbf{u}_{i,\mathbf{k}}) = i\int_{\mathrm{V}} \varepsilon \mathbf{u}_{i,\mathbf{k}}^{*} \nabla_{\mathbf{k}} \mathbf{u}_{i,\mathbf{k}} \mathrm{d}^3\mathbf{r}, \tag{51}$$

in which $\mathbf{u}_{i,\mathbf{k}}$ (or $\mathbf{u}_i(\mathbf{k})$) is the periodic part of the electric field $\mathbf{E}_i(\mathbf{k})$ of the state. Then the Berry curvature (z component) is

$$\Omega = \frac{\partial B_x}{\partial y} - \frac{\partial B_y}{\partial x}. \tag{52}$$

When numerically calculating the Berry curvature at a certain point A(k_x, k_y) in the reciprocal space, we consider a clockwise small square contour around A consisting of four points $\mathrm{A}_1(k_x-\delta k/2,\ k_y-\delta k/2)$, $\mathrm{A}_2(k_x-\delta k/2,\ k_y+\delta k/2)$, $\mathrm{A}_3(k_x+\delta k/2,\ k_y+\delta k/2)$, and $\mathrm{A}_4(k_x+\delta k/2,\ k_y-\delta k/2)$ as depicted in Supplementary Fig. 12. For convenience, the region enclosed by the square contour with the side length δk is denoted as S and the contour itself is denoted as ∂S. Then directly utilizing Stokes' theorem, we have

$$\int_{\mathrm{S}} \Omega \mathrm{d}^2\mathbf{k} = -\int_{\partial \mathrm{S}} \mathbf{B} \cdot \mathrm{d}\mathbf{k}, \tag{53}$$

in which each side could be discretized and estimated as[9]

$$\int_{\mathrm{S}} \Omega \mathrm{d}^2\mathbf{k} = \Omega(\mathrm{A})(\delta k)^2, \tag{54}$$

$$\begin{aligned}\int_{\partial \mathrm{S}} \mathbf{B} \cdot \mathrm{d}\mathbf{k} = -\,\mathrm{Im}[&(\mathbf{u}_i(\mathrm{A}_1), \mathbf{u}_i(\mathrm{A}_2)) + (\mathbf{u}_i(\mathrm{A}_2), \mathbf{u}_i(\mathrm{A}_3)) \\ &+ (\mathbf{u}_i(\mathrm{A}_3), \mathbf{u}_i(\mathrm{A}_4)) + (\mathbf{u}_i(\mathrm{A}_4), \mathbf{u}_i(\mathrm{A}_1))],\end{aligned} \tag{55}$$

where (,) is the inner product defined in Eq. (1). The two estimations in Eqs. (54) and (55) can be justified when δk is much smaller than the side length of the FBZ. In our case, δk = 1 m^{-1} and the side length of the FBZ is 349.1 m^{-1}, hence the estimations are reasonable. Then we have the following expression used for numerical calculations

$$\begin{aligned}\Omega(\mathrm{A}) = \mathrm{Im}[&(\mathbf{u}_i(\mathrm{A}_1), \mathbf{u}_i(\mathrm{A}_2)) + (\mathbf{u}_i(\mathrm{A}_2), \mathbf{u}_i(\mathrm{A}_3)) \\ &+ (\mathbf{u}_i(\mathrm{A}_3), \mathbf{u}_i(\mathrm{A}_4)) + (\mathbf{u}_i(\mathrm{A}_4), \mathbf{u}_i(\mathrm{A}_1))] / (\delta k)^2,\end{aligned} \tag{56}$$

in which the inner products of the electric fields are calculated in COMSOL

Multiphysics using the function of volume integration.

Supplementary Note 5

One-way Propagation of Valley-polarized Edge States

Inspired by a previous work[2], we use a domain wall terminated by zigzag edges to demonstrate one-way propagation of valley-polarized edge states because these edge states are immune from backscattering at zigzag edges. Further, in order to excite valley-polarized edge states in the middle of domain walls, we use three phase-matched electrical dipoles[2] which are slightly staggered to avoid directly contacting with radials fans of the metallic patterns, and the phase difference between neighboring dipoles are tuned such that they are matched with the phase of an edge state at the K' valley. As a result, the valley-polarized edge states of AB-type and BA-type domains are successfully excited and the field maps of their E_z component are shown in Supplementary Figs. 5(a), and 5(b), respectively. One-way propagation, a manifestation of valley-locked chirality, can be clearly seen from the field maps.

We then did experiments using the same scheme. Three monopole antennas with the same length were attached at the center of the fabricated samples. The monopole antennas were fabricated by tailoring thin co-axial lines. Three phase shifters connected to a power divider were one-to-one connected to the three monopole antennas to control their phases. Near-field scanning was then performed and the obtained field maps are shown in Supplementary Fig. 6, from which one-way propagation of the edge states could also be observed.

Supplementary Note 6

Measurement of Background Fields

When measuring the background fields, we use a dielectric sample of the same geometric shape of the beam splitter, but with no metallic patterns. The source is attached at the same position of terminal 1 or terminal 2 of the beam splitter, and then we scan the same region which is shown in Fig. 5(c) or 5(d). The scanned background field is shown in Supplementary Fig. 8(c) and 8(d), and we also show the scanned fields when there are metallic patterns in Supplementary Fig. 8(a) and 8(b) for comparison. Then we performed spatial Fourier transforms on the measured background fields and the corresponding spatial Fourier spectra in Supplementary Fig. 8(e) and 8(f) only show faint regions on the light cone, suggesting that the measured fields are only EM waves propagating in air. Similar faint regions on the light cone are observed in Fig. 5(e) and 5(f), hence confirming that the measured "inter-valley" transports (t_{31} and t_{42} in Fig. 4) are largely owing to the uncoupled EM wave in air.

Supplementary References

1 Wang, H., Xu, L., Chen, H. & Jiang, J.-H. Three-dimensional photonic Dirac points stabilized by point group symmetry. *Physical Review B* **93**, 235155 (2016).

2 Ma, T. & Shvets, G. All-Si valley-Hall photonic topological insulator. *New Journal of Physics* **18**, 025012 (2016).

3 Mei, J., Wu, Y., Chan, C. & Zhang, Z.-Q. First-principles study of Dirac and Dirac-like cones in phononic and photonic crystals. *Physical Review B* **86**, 035141 (2012).

4 Joannopoulos, J. D., Johnson, S. G., Winn, J. N. & Meade, R. D. *Photonic crystals: molding the flow of light.* (Princeton University Press, 2011).

5 Morpurgo, A. & Guinea, F. Intervalley scattering, long-range disorder, and effective time-reversal symmetry breaking in graphene. *Physical Review*

Letters **97**, 196804 (2006).

6 Lu, J. *et al.* Observation of topological valley transport of sound in sonic crystals. *Nature Physics* **13**, 369-374 (2016).

7 Tkachov, G. *Topological insulators: The physics of spin helicity in quantum transport*. (CRC Press, 2015).

8 Wang, Z., Chong, Y., Joannopoulos, J. & Soljačić, M. Observation of unidirectional backscattering-immune topological electromagnetic states. *Nature* **461**, 772-775 (2009).

9 Fukui, T., Hatsugai, Y. & Suzuki, H. Chern numbers in discretized Brillouin zone: Efficient method of computing (spin) Hall conductances. *Journal of the Physical Society of Japan* **74**, 1674-1677 (2005).
